# Approaches in analyzing predictors of trial failure: a scoping review and meta-epidemiological study

**DOI:** 10.1186/s12874-026-02774-8

**Published:** 2026-01-17

**Authors:** Aleksa Jovanovic, Stojan Gavric, Fabio Dennstädt, Nikola Cihoric

**Affiliations:** 1 Wemedoo AG, Sumpfstrasse 24, Steinhausen, 6312 Switzerland; 2 Department of Radiation Oncology, Inselspital, Bern University Hospital, University of Bern, Bern, Switzerland

**Keywords:** Trial termination, Trial attrition, Trial discontinuation, Systematic review, Study design, Statistical methods, Risk factors

## Abstract

**Background:**

Although there are numerous studies exploring predictors of clinical trial failure, no comprehensive review of their methodological specificities and findings exists. We performed a scoping review with the aim of exploring the methodological approaches and findings of studies analysing predictors of clinical trial failure.

**Methods:**

The Ovid Medline and Embase databases were systematically searched from inception to December 13, 2024, for studies employing frequentist statistics or machine learning (ML) approaches to assess predictors of trial failure across multiple clinical trials. A generalized linear model (GLM) was employed to assess the impact of certain methodological factors (failure and non-failure definitions, study types included and trial phases included) on reported failure proportions. To estimate the effects of the predictors included in the model on failure proportions, odds ratios (OR) with 95% confidence interval (95% CI) were calculated from model coefficients.

**Results:**

The literature search identified 17,961 records, 81 of which were included in the review. Most of the studies used Clinicaltrials.gov data (73 studies, 90.1%). Frequentist statistics were used to analyze predictors of trial failure in 73 studies (90.1%), and remaining 8 studies employed ML techniques (9.9%). The GLM showed a 27.5% deviance reduction, indicating that certain methodological factors substantially contribute to observed differences in failure proportions. Studies including trials with both completed and ongoing statuses when calculating failure proportions had lower odds of failure compared to those just including completed statuses (OR = 0.44, 95% CI: 0.29–0.67, *p* < 0.001).

**Conclusions:**

There has been a recent expansion of ML approaches, potentially signaling the beginning of a paradigm shift. Methodological variations substantially influence reported failure proportions, implicating the need for adoption of standardized definitions of failure and calculation approach. We recommend categorizing terminated and withdrawn studies as failed and completed ones as non-failed.

**Supplementary Information:**

The online version contains supplementary material available at 10.1186/s12874-026-02774-8.

## Background

Understanding what constitutes a failed clinical trial requires clarifying a common misconception about negative results. Contrary to popular belief, trials with negative results should not be considered failed, since they contribute to the scientific knowledge base [1]. Furthermore, refusal to publish these negative results leads to publication bias which can later have negative implications on more comprehensive knowledge synthesis such as meta-analyses [2, 3]. On the other hand, prematurely terminated clinical trials can be considered failed, as they result in a loss of opportunity for expanding the knowledge base. Premature termination (i.e. clinical trial failure) also has other direct and indirect consequences on the healthcare and scientific ecosystem, such as resource depletion and loss of motivation to reevaluate the same disease or interventions [4].

To prevent these consequences, a growing body of literature employs statistical methods to identify predictors of trial failure. However, the validity of these analyses rests entirely on how the outcome itself is constructed. A fundamental prerequisite for identifying risk factors is the binary classification of trials into “failures” and “non-failures”. Currently, there is no standardized framework or consensus regarding which trial statuses constitute the failure event (numerator) and which comprise the reference group (denominator). This methodological ambiguity makes it difficult to interpret the literature: when failure estimates and predictors vary significantly across therapeutic areas, it remains unclear whether these differences reflect intrinsic medical specificities or are simply artifacts of the varying definitions employed.

Therefore, we performed a scoping review and meta-epidemiological study with the aim of exploring the methodological approaches and findings of studies systematically analysing predictors of trial failure. Specifically, we aimed to: (1) Systematically map the methodological factors used in current research, including failure and non-failure definitions, eligibility criteria, and statistical approaches; (2) Quantify the influence of these methodological factors on reported failure proportions in studies analyzing different therapeutic areas; and (3) Synthetize the reported findings (failure proportions and statistically significant failure predictors) of the included studies.

## Methods

### Information sources and search strategies

We performed a scoping review according to the Preferred Reporting Items for Systematic Reviews and Meta-Analyses Extension for Scoping Reviews (PRISMA-ScR) Guidelines [5] (eTable 1). The Ovid Medline and Embase databases were searched from inception to December 13, 2024. The complete search strategy for each of the databases is provided in eTables 2 and 3. An e-mail was sent to the corresponding author when the full text of a study fitting the inclusion criteria was unavailable.

### Eligibility criteria

Studies were included if they employed frequentist statistics or machine learning (ML) approaches to systematically assess predictors of trial failure (premature termination) across multiple clinical trials. We included cross-sectional, case-control, and modelling studies performing retrospective analyses of clinical trial registry data. No restrictions were placed on therapeutic area, demographics, publication date, language or type. Research articles, letters to the editor and conference abstracts all were deemed eligible if they fit the scope of the review. In the case of overlapping publications from the same research group, the most comprehensive report was included.

Studies were excluded if they: (1) presented predictor distributions without statistical analysis; (2) summarized existing literature without analysis; (3) analyzed predictors of trial failure due to isolated reasons such as failed accrual; 4 ) examined predictors non-systematically across only 1–2 trials; 5) were individual trial failure reports; 6) studied animal or cell models.

### Study selection

Recent research has shown that large language models (LLMs) can achieve high sensitivity in screening scientific publications for systematic reviews, with sensitivity rates of over 97% for certain models shown [6, 7]. The study selection process utilized the claude-3-5-sonnet-20,241,022 over Anthropic API LLM for initial screening of titles and abstracts. We employed a two-step approach, with an additional human check in step two, achieving 100% sensitivity and ~ 84% specificity in each of the steps (eFigure 1). In the first step, the LLM excluded the papers whose titles were clearly unrelated to any aspect of trial failure. In the second step, it categorized the abstracts of the remaining papers into five relevance levels. A human reviewer then assessed the titles of studies ranked as categories 2 or 3 and read the full abstracts of those ranked 4 or 5. Full details on the validation process and prompts used in the screening are presented in eAppendices 1 and 2, respectively. After screening titles and abstracts, the full text of papers assessed as relevant was obtained and assessed independently by two reviewers (A.J. and S.G.). The disagreements in this phase were resolved by consensus after involving the third reviewer (N.C).

### Data charting and data items

A data charting form was developed jointly by two reviewers (A.J. and S.G.), who independently charted data. The included studies were assessed for the: (a) study meta-data (authors, title, year of publication), (b) presence of results reporting and/or non-publication analysis, (c) data source, (d) therapeutic area, (e) methodological factors [eligibility criteria (study type, phase, demographics, trial location restrictions, etc.), failure definitions, non-failure definitions (i.e. the categories used as the denominator for computation of failure proportion), failure predictors assessed, statistical approach used for assessing failure predictors], (f) number of trials analyzed and (g) findings (proportion of failed studies based on the definitions used, distribution of failure reasons, statistically significant failure predictors in studies employing frequentist statistics). For studies employing ML approaches we additionally extracted the types of structured and unstructured features used, feature engineering techniques, ML algorithms, evaluation metrics results, comparison to traditional methods, interpretability methods employed.

For studies using Clinicaltrials.gov datasets, we grouped some of the study statuses into broader categories: (1) Active: Included studies listed as “recruiting”, “enrolling by invitation”, or “active, not recruiting”; (2) Ongoing: Included all Active statuses (“recruiting”, “enrolling by invitation”, or “active, not recruiting”), as well as “not yet recruiting” status. When studies didn’t explicitly state which Clinicaltrials.gov definitions were used for failure proportion calculation, we made assumptions based on their methods and results section. In rare instances where a study used a different number of studies for predictor analysis and failure proportion calculation, we used the number related to failure proportions to maintain consistency.

During extraction of frequentist statistical methods, chi-square was extracted if it was the sole analysis conducted to explore associations between trial-level factors and trial failure, but wasn’t extracted when performed as a supplementary analysis to another, more sophisticated analyses (i.e. regression model). The exception was when the authors explicitly stated that the statistical significance of chi-square test was used to select variables for inclusion in the multivariable model.

### Synthesis of results

Therapeutic areas were grouped into broader categories (e.g. neuro-oncology was grouped into oncology category) when presenting descriptives. Descriptive statistics were used to calculate summary statistics of key characteristics of included studies.

A matrix of failure proportion distribution according to combinations of failure and non-failure definitions was computed for studies utilizing data from Clinicaltrials.gov (eAppendix 3). The analysis was repeated in two subsets of studies including only: (a) interventional studies (both randomized and non-randomized controlled trials); (b) randomized controlled trials (RCTs).

### Statistical analysis

Supplementary to our scoping review, we employed a generalized linear model (GLM) to quantify the extent to which certain methodological factors (failure and non-failure definitions, study types included and trial phases included) contribute to observed differences in failure proportions in studies analyzing different therapeutic areas. Therapeutic area couldn’t be incorporated as a variable in the model since the analyzed studies shared a common data source (Clinicaltrials.gov), which meant that certain trials would be counted multiple times across different studies, while others would appear only once. We avoided this bias by stratifying studies based on the therapeutic area and then selecting the most representative ones from each subset. A detailed explanation on the selection process of studies for the GLM, variable categorization and model selection are presented in eAppendix 4.

To estimate the effects of the predictors included in the model on failure proportions, odds ratios (OR) with 95% confidence interval (95% CI) were calculated from model coefficients. Significance level was set at *p* < 0.05. Analyses were conducted in R (version 4.3.1).

## Results

### Study selection

The literature search identified 17,961 records from Ovid Medline and Embase databases. After duplicate removal, the title and abstract screening phase was performed in multiple stages (eFigure 2), leading to 111 papers being assessed for eligibility, of which 93 were full papers, and 18 were conference abstracts. After assessing full manuscripts and conference abstracts for relevance, 81 studies [8–88] were finally chosen for inclusion (eFigure 2). Of these, 7 were reports from a conference, and for a single paper the full text couldn’t be obtained even after contacting the authors, so only the abstract could be included. Due to ambiguity of failure and non-failure definitions and limited comprehensiveness of the data available in these 8 abstracts, they were excluded from failure classification analyses and the GLM analysis.

### Characteristics of included studies

Most of the 81 included studies used Clinicaltrials.gov data (73 studies, 90.1%) (Table 1). More than a third of included papers (32, 39.5%) assessed predictors for non-publication in addition to predictors of premature termination, while a minority (11, 13.6%) assessed the predictors of results reporting.

**Table 1:** Distribution of characteristics of included studies

Study characteristic	All studies (N=81) Studies, No. (%)	Full text studies analysing Ct.gov (N=66) Studies, No. (%)
Data source		
Ct.gov	73 (90.1)	66 (100.0)
Other national databases	5 (6.2)	0 (0.0)
EudraCT	2 (2.5)	0 (0.0)
Medline	1 (1.2)	0 (0.0)
Topic		
Oncology	21 (25.9)	19 (28.8)
Surgery	13 (16.1)	13 (19.7)
Internal medicine	9 (11.1)	4 (6.1)
No restriction	15 (18.5)	9 (13.6)
Neurology	5 (6.2)	4 (6.1)
Obstetrics and gynecology	4 (4.9)	4 (6.1)
Pediatrics	4 (4.9)	3 (4.5)
Psychiatry	3 (3.7)	3 (4.5)
Other	7 (8.7)	7 (10.6)
Study type included		
Any	16 (19.7)	12 (18.2)
Interventional only	43 (53.1)	35 (53.0)
RCT only	22 (27.2)	19 (28.8)
Failed study definition	NA	
Terminated, Withdrawn or Suspended		31 (47.0)
Terminated		12 (18.2)
Terminated or Withdrawn		10 (15.1)
Terminated, Withdrawn, Suspended or Unknown		6 (9.1)
Other 6 definitions		7 (10.6)
Non-failed study definition	NA	
Completed		38 (57.6)
Completed, Ongoing*, Unknown		12 (18.2)
Other 12 definitions		16 (24.2)

Legend: *Ongoing entails Recruiting, Enrolling by invitation, Active, not recruiting, and Not yet recruiting statuses. NA: Not applicable. Statistics for the full sample (N=81) are not calculate due to the heterogeneity and ambiguity of definitions in the 15 studies (including abstracts and non-ClinicalTrials.gov sources) excluded from the detailed classification analysis. Recruitment status definitions (ClinicalTrials.gov): 1) Not yet recruiting: The study has not started recruiting participants; 2) Recruiting: The study is currently recruiting participants; 3) Enrolling by invitation: The study is selecting its participants from a population, or group of people, decided on by the researchers in advance. These studies are not open to everyone who meets the eligibility criteria but only to people in that particular population, who are specifically invited to participate; 4) Active, not recruiting: The study is ongoing, and participants are receiving an intervention or being examined, but potential participants are not currently being recruited or enrolled; 5) Suspended: The study has stopped early but may start again; 6) Terminated: The study has stopped early and will not start again. Participants are no longer being examined or treated; 7) Completed: The study has ended normally, and participants are no longer being examined or treated (that is, the last participant's last visit has occurred); 8) Withdrawn: The study stopped early, before enrolling its first participant; 9) Unknown: A study on ClinicalTrials.gov whose last known status was recruiting; not yet recruiting; or active, not recruiting but that has passed its completion date, and the status has not been last verified within the past 2 years [89]

The most frequently analyzed major therapeutic area was oncology (21, 25.9%), followed by studies analyzing the dataset comprehensively, without restrictions on the therapeutic area or demographics (15, 18.5%) (Table 1). Seven of the studies (8.7%) each analyzed a unique therapeutic area (Table 1).

Most studies excluded observational studies from the analysis (65, 80.2%), while about a quarter of the total studies (22, 27.2%) focused exclusively on RCTs, also excluding non-randomized clinical trials (Table 1). Across the included studies, the number of clinical trials analyzed per study had a median of 746, with counts ranging from 54 to 284,644 (eTable 4). The median failure proportion in all included studies was 18%, ranging from 3% in a study analyzing 312 drug clinical trials registered in China, to 51.8% in a study analyzing 85 cervical cancer and precancer trials registered at Clinicaltrials.gov (eTable 4).

### Clinical trial failure classifications

The trial failure proportions are calculated as the number of failed studies over the total number of studies analyzed (i.e. the sum of failed and non-failed studies). We explored the differences in definitions of failure and non-failure across studies to examine whether they influence the failure proportions. There were 10 distinct clinical trial failure definitions among the 66 full text papers analyzing studies registered in Clinicaltrials.gov (eTable 4), with almost half of the studies defined failure as terminated, withdrawn or suspended trial status (31 studies, 47.0%) (Table 1). When considering non-failure definitions, the diversity was even greater. There were 14 distinct definitions among the 65 papers (eTable 4), with more than half of the papers including only the completed status (38 studies, 57.6%) (Table 1).

**Fig. 1:**
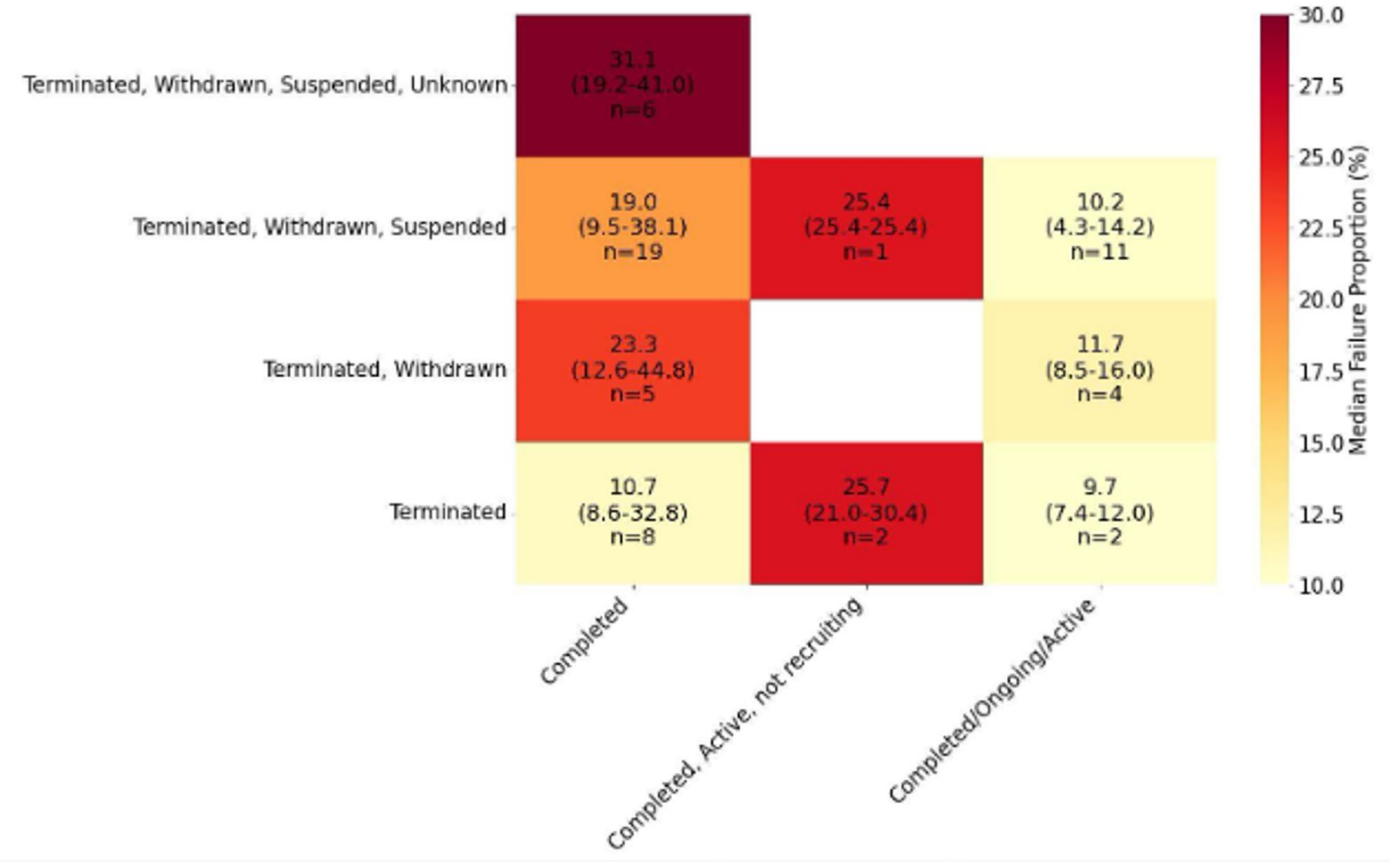
Failure proportion distribution by trial failure and non-failure definitions, median (range)

Failure proportions varied depending on the combination of failure and non-failure definitions used (Fig. 1). After merging categories and discarding unique categories (eAppendix 3), 57 studies with 4 distinct failure and 3 distinct non-failure definitions for a total of 9 combinations were left in the analysis (Fig. 1). The lowest failure proportions were observed in the two studies that defined failure as terminated and counted completed plus ongoing/active trials as non-failed; the failure proportions reported in these studies had a median of 9.7% (range 4.3–14.2) [36, 54]. In contrast, the highest failure proportions were found in studies defining failure as terminated, withdrawn, suspended, or unknown, and defining non-failure as completed only, with reported failure proportions showing a median of 31.1% (range 19.2–41.0) [11, 18, 31, 75–77]. The distribution of failure proportions according to failure and non-failure definitions in studies excluding observational trials is shown in eFigure 3, and in studies including only RCTs is presented in eFigure 4.

### Influence of methodological factors on observed failure proportions

The beta-binomial model provided a better fit compared to the null model (AIC = 237.06 vs. 250.66). The model achieved a 27.5% reduction in null deviance (from 100.26 to 72.66), indicating that methodological factors contribute substantially to observed differences in failure proportions.

Non-failure definition and study type were statistically significant predictors of failure proportions in the beta-binomial model. Studies including both completed and ongoing trials when calculating failure proportion had lower odds of failure compared to those just including trials with completed status as non-failures (OR = 0.44, 95% CI: 0.29–0.67, *p* < 0.001). Studies including strictly randomized trials had higher odds of failure than studies including both randomized and non-randomized trials (OR = 1.89, 95% CI: 1.12–3.19, *p* = 0.016). On the other hand, studies including both observational and interventional studies had lower odds (OR = 0.53, 95% CI: 0.34–0.85, *p* = 0.007) of failure compared with studies including only interventional studies (both randomized and non-randomized trials). The failure definitions and trial phases included weren’t statistically significant predictors of failure proportions.

### Statistical approaches in assessing failure predictors

**Fig. 2:**
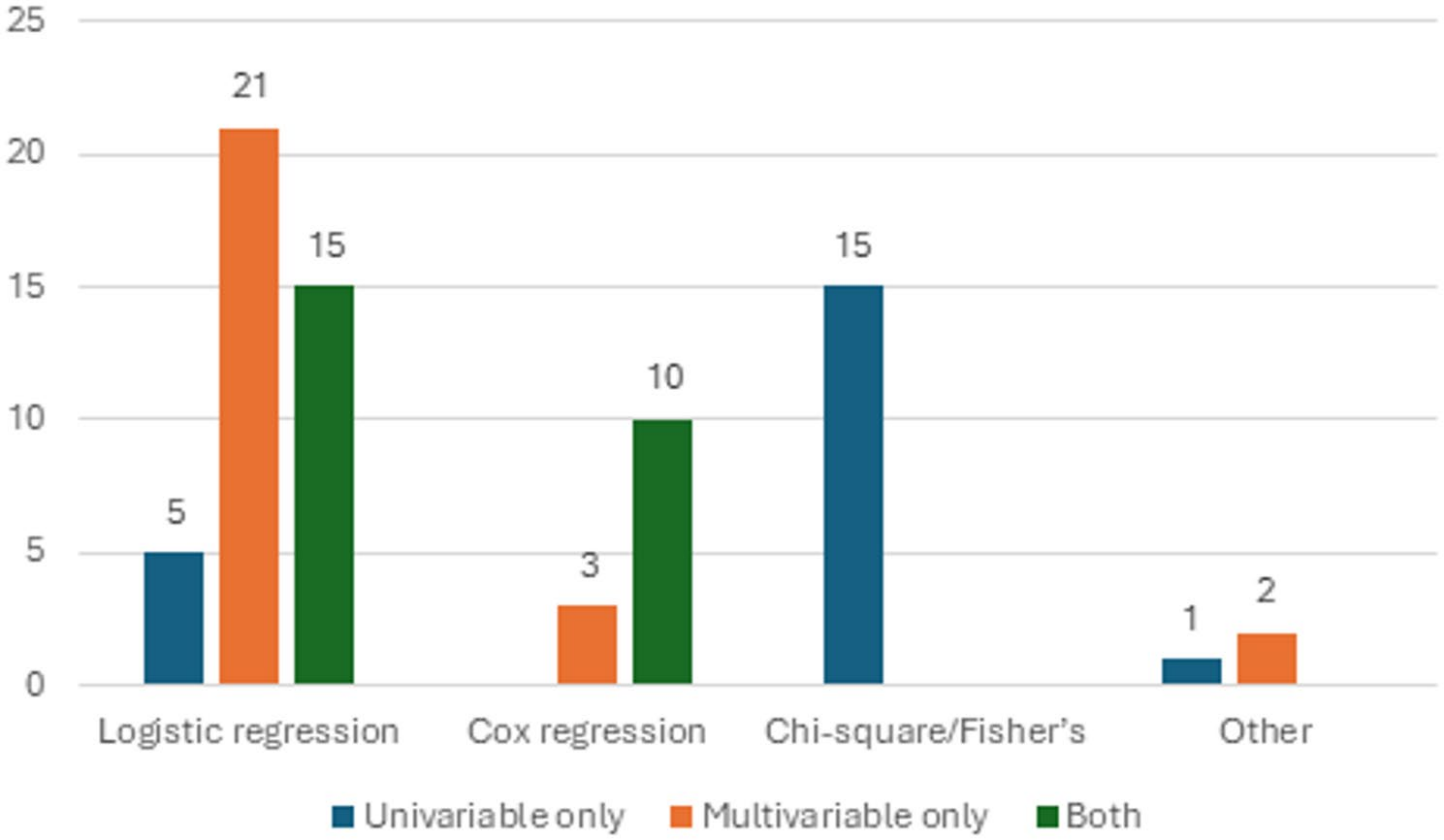
Distribution of frequentist statistics methodologies employed for predictor analysis

All but eight studies identified in the review (73 studies, 90.1%) used frequentist statistics to assess predictors of trial failure. Among these, one conference abstract stated that “hazard ratio analysis” was performed, but results presented ORs, prompting us to exclude the study from the distribution analysis due to ambiguity. Of the remaining 72 studies employing frequentist statistics, logistic regression was most frequently used (41 studies, 56.9%), followed by chi-square/Fisher’s exact test (15 studies, 20.8%), and Cox regression analysis (13 studies, 18.1%). One study employed multivariable Poisson regression, one employed GLM, one claimed that “relative risks” were calculated without stating which test was used. Almost three quarters (52 studies, 72.2%) of studies employing frequentist statistics employed a multivariable analysis controlling for confounding. Two studies utilizing multivariable logistic regression chose the predictors to include in the multivariable model based on the significance of chi-square test (Fig. 2).

Eight studies employed ML approaches to analyze predictors of trial failure, with the first study published in 2019, and half of the studies published in 2023. Approaches ranged from conventional statistical learning to modern neural architectures (Table 2). Four studies used established techniques such as LASSO, Random Forest, gradient boosting, while others employed more specialized approaches: Latent Dirichlet Allocation (LDA) for topic extraction from trial protocols by using probabilistic topic modeling, neural networks for survival analysis, and transformer-based language models for text processing.

**Table 2:** Methodological characteristics of machine learning papers

Author	New feature engineered features	Free-text fields used from CT.gov	Feature engineering techniques	Machine learning algorithms	Evaluation metrics results for the	Comparison to traditional methods	Interpretability methods
Lee et al. 2023 [15]	No	None	MICE, LASSO regularization for variable selection	LASSO, Elastic Net, Ridge Regression, Random Forest, AdaBoost Classification Trees, GLMboost, BART, Super Learner (an ensemble method combining the above approaches)	LASSO - AUROC: 0.70 (95% CI: 0.53–0.86), Sn: 0.70 (95% CI: 0.28–0.91), Sp: 0.66 (95% CI: 0.35–0.90), PPV: 0.56 (95% CI: 0.41–0.71), NPV: 0.76 (95% CI: 0.65–0.87)	None	LASSO coefficients
Kavalci et al. 2023 [35]	Yes	Eligibility criteria	ANOVA F-score based feature selection, n-gram language model (5-gram)	XGBoost, Random Forest, Logistic Regression	XGBoost - ROC AUC: 79.78%, balanced accuracy: 70.22%, F1 score (class 0): 41.80%, class 0 accuracy: 70.70%, class 1 accuracy: 69.70%	Logistic Regression	SHAP
Geletta et al. 2019 [37]	No	Brief summary	Tokenization, Document-term matrix, LDA topic modeling, Topic selection	Random Forest, Logistic Regression (post-hoc)	Random Forest with LDA-derived topics - ROC analysis: Sn (at 0.3 FP rate): 0.6, Sn (at 0.5 FP rate): 0.8	None	Model-native interpretability methods
Kim et al. 2022 [41]	Yes	None	Categorization, Time-to-Event processing	Cox proportional hazard model (with stepwise feature selection), DeepSurv (a neural network for survival analysis)	DeepSurv - Concordance index: 0.72, SE = 0.003	Cox proportional hazard model	Cox proportional hazard model, nomogram, concordance index
Elkin et al. 2021a [42]	Yes	Eligibility criteria, Keywords	TF-IDF, Doc2Vec	Neural Network, Random Forest, XGBoost, Logistic Regression	Ensemble XGBoost - AUC: 0.73, balanced accuracy: 67.20%, F1-Score: 31.21%	Logistic Regression	Feature ranking techniques
Elkin et al. 2021b [57]	Yes	Detailed Description, Keywords	TF-IDF, Doc2Vec, Domain-specific engineering	Neural Network, Random Forest, XGBoost, Logistic Regression	Ensemble Random Forest - AUC: 0.87, balanced accuracy: 0.81, F1-score: 0.60	None. Logistic regression was treated as part of the ML suite	ReliefF feature ranking, Feature type importance analysis
Chang et al. 2023 [58]	Yes	Eligibility criteria	Discretization, Consolidation, NLP processing using Clinical-Trials-Parser, Structured representation	Contrast pattern mining, Lasso Logistic Regression, Random Forest	No single model singled out as being superior, contrast pattern classifier achieved around 80% F1 score for 8 of 10 cancer types	Lasso Logistic Regression, Random Forest	Contrast pattern mining
Luo et al. 2023 [64]	No	Failure reasons	Dense embedding transformation, text aggregation	LSTM, XLNet, DistilBERT, BERT, Longformer, MultiResCNN, HAN, GCN-selective, DistilBERT-Concat, DistilBERT-Average	DistilBERT-Concat Status prediction accuracy: 0.707, AUC: 0.777; Failure Reason Prediction accuracy: 0.726, Micro F1: 0.413, Macro F1: 0.185	None	None

Abbreviations: *CT.gov* ClinicalTrials.gov, *ML* Machine learning, *MICE* Multivariate imputation by chained equations, *LASSO* Least absolute shrinkage and selection operator, *GLMboost* Boosted Generalized Linear Regression, *BART* Bayesian Additive Regression Tree, *PPV* Positive Predictive Value, *NPV* Negative Predictive Value, *AUROC* Area Under Receiver Operating Characteristic, *Sn* Sensitivity, *Sp* Specificity, *LDA* Latent Dirichlet Allocation, *ROC* receiver operating characteristic, *OR* Odds ratio, *FP *false-positive, *ANOVA* Analysis of Variance, *XGBoost* Extreme gradient boosting classifier, *AUC* Area Under the Curve, *SHAP* Shapley Additive Explanations, *SE* standard error, *MeSH* Medical Subject Headings, *TF-IDF* Term Frequency-Inverse Document Frequency, *Doc2Vec* Document-to-Vector, *NLP* Natural Language Processing, *GLM* Generalized Linear Models, *LSTM* Long Short-Term Memory, *XLNet* eXtreme Learning Net, *DistilBERT* Distilled Bidirectional Encoder Representations from Transformers, *BERT* Bidirectional Encoder Representations from Transformers, *Longformer* Long Document Transformer, *MultiResCNN* Multi-Resolution Convolutional Neural Network, *HAN* Hierarchical Attention Network, *GCN-selective* Graph Convolutional Network (Selective), *DistilBERT-Concat* Distilled Bidirectional Encoder Representations from Transformers (Concatenation), *DistilBERT-Average* Distilled Bidirectional Encoder Representations from Transformers (Averaging)

### Trial failure predictors

Table 3 shows the distribution of results of multivariable analyses analyzing predictors of trial failure. There were two factors that were shown to be statistically significant predictors of trial failure in more than 50% studies which assessed them: smaller enrollment (found to be predictive of trial failure in 18/25 studies [9, 18, 23, 29, 37, 38, 50, 53–56, 59, 63, 65, 69, 71, 79, 85]) and US-based trials (10/18 studies [15, 30, 43, 53, 56, 60, 63, 70, 76, 87]). The statistically significant findings from multivariable analysis of failure predictors from included studies are presented in eTable 5, with a detailed summary provided in eAppendix 5.

**Table 3:** Predictors of trial failure

Predictor	Studies analyzing the predictor Studies, No. (%)	Failure risk factor Studies, No. (%)	Protective of failure Studies, No. (%)	Non-significant Studies, No. (%)
Enrollment	25 (100)	18 (72)	0 (0)	7 (28)
Anticipated accrual	8 (100)	1 (12.5)	2 (25)	5 (62.5)
Location (US)	18 (100)	10 (55.6)	0 (0)	8 (44.4)
Higher number of participating countries	3 (100)	1 (33.3)	0 (0)	2 (66.7)
Higher number of participating centers	21 (100)	5 (23.8)	3 (14.3)	13 (61.9)
Blinding	23 (100)	3 (13)	3 (13)	17 (73.9)
Randomization	16 (100)	2 (12.5)	0 (0)	14 (87.5)
Presence of a data monitoring committee	3 (100)	1 (33.3)	1 (33.3)	1 (33.3)

## Discussion

Our scoping review revealed significant differences in defining failure and selecting trial statuses for denominators, contributing to wide variation in reported failure proportions. The extremes of the failure proportions illustrate how denominator selection impacts its value. The five studies with the highest failure proportions (40.1%–46.1%) all used restrictive denominators (i.e., only completed trials as non-failures, excluding the ongoing/active trials from the total count) but differed in failure definitions (all included terminated and withdrawn, some additionally included suspended and/or unknown) [11, 15, 64, 80, 85]. Additionally, some of these studies had important specificities further inflating the failure proportions: counting only studies with published results as completed [80]; not including all completed trials because of implementation of a 1:1 case-control design [64]; or categorizing studies completed with < 85% of targeted sample size as failed [15]. The five studies had widely different scope (urology, therapy-related, cancer trials in older adults, neuro-oncology, and extremity fracture), indicating that the investigated therapeutic area isn’t the only driver of differences between failure proportions.

Furthermore, the studies reporting the lowest failure proportions (4.3% and 5.6%) included all 8 Clinicaltrials.gov statuses in the denominator, thereby diluting the failure proportions [36, 54]. Including ongoing trials when reporting failure proportions underestimates the true failure proportion since some of these trials will ultimately fail, but none of them are counted as failed since, at present day, their outcomes are still unknown. Analyzing predictors of failure while including ongoing studies can lead to misleading implications, since these studies have systematically different trial characteristics compared to completed ones [90]. Similarly, a common practice of counting suspended trials as failed should be challenged, since these trials, by definition, could restart and complete successfully [89]. We propose standardizing definitions for future research, recommending categorizing terminated and withdrawn studies as failed and only completed ones as non-failed. This approach would provide more accurate measurements of prevalence and predictors of trial failure by eliminating the confounding of studies that still have the potential to be completed either successfully or unsuccessfully.

Another methodological difference influencing the observed failure proportions is the type of studies included in the analysis. This is in line with the association observed in several studies that randomization is associated with trial failure [51, 68, 75, 88]. Analyses restricted to RCTs showed 90% higher failure odds compared to those including both randomized and non-randomized interventional trials. This suggests might RCTs face greater completion challenges due to their more rigorous methodological requirements, although the findings from the multivariable analyses for randomization as the risk factor for failure were not convincing [9, 12, 13, 16, 22, 29, 30, 38, 41, 50, 52–54, 65, 79, 80]. On the other hand, studies including observational trials showed lower failure odds (OR = 0.52, 95% CI: 0.33–0.82), consistent with the generally simpler execution of observational studies [27, 51, 56, 72].

In our review, we identified eight studies that have employed ML techniques to analyze predictors of trial failure [15, 35, 37, 41, 42, 57, 58, 64]. This novel approach has several advantages over traditional statistical methods. First, it can handle a much larger number of features, and collinearity and non-linearity are not issues as in traditional statistical models [91]. Second, the ML approach has the option to automatically detect feature interactions. Third, it has the ability to handle unstructured data (e.g., trial protocols, eligibility criteria) [92]. Additionally, by employing interpretability techniques, it can rank the relative importance of different predictors in the model’s decision. As opposed to summary statistics used in traditional statistics, these interpretability techniques can be implemented to give a risk profile based on the relative importance of different features integrated into the model on a study-by-study basis [93]. This is important for clinical trials’ stakeholders, as it provides transparency needed to justify potential protocol alterations. Further development and implementation of these models could lead to reducing clinical trial failure proportions.

Our study has several limitations. First, although we implemented a multi-iterative LLM-assisted screening approach and validated it showing 100% sensitivity, the possibility remains that some relevant studies were missed. However, due to the vast number of correctly identified and included studies in the review, and the absence of false negatives in categories 2 or 3 in the more complex abstract screening step, we deem it highly unlikely. Second, our analysis of methodological factors affecting failure proportions was limited by the potential overlap in trials, forcing us to exclude several studies with larger sample sizes from modelling. This also prevented us from including therapeutic area in the model to provide an estimate on how much of the trial failure proportion variation can be attributed to it. Finally, the specific impact of many rare non-failure definitions could not be analyzed, due to lack of studies. However, our model encompassed the most important and the most frequent categories.

## Conclusions

Our findings show the limitations of comparing failure proportions across therapeutic areas by comparing calculated proportions across studies. A need exists for adoption of standardized definitions of trial failure and non-failure for more accurate comparisons. We recommend categorizing terminated and withdrawn studies as failed and solely completed ones as non-failed. Finally, the recent expansion of ML models for predicting trial failure shows a promising prospect for reducing clinical trial failure in the future.

## Supplementary Information

Supplementary Material 1.

## Data Availability

The data sets used and/or analyzed during the study are available from the corresponding author on request.
